# Potentially inappropriate prescribing to older patients receiving multidose drug dispensing

**DOI:** 10.1186/s12877-020-01665-x

**Published:** 2020-08-05

**Authors:** Anette Vik Josendal, Trine Strand Bergmo, Anne Gerd Granas

**Affiliations:** 1 Norwegian Centre for E-health Research, University Hospital of North Norway, Tromsø, Norway; 2 Department of Pharmacy, Section for Pharmaceutics and Social Pharmacy, University of Oslo, Oslo, Norway

**Keywords:** Multidose drug dispensing, Inappropriate prescribing, Elderly, Norway, Home care services, Drug-drug interactions

## Abstract

**Background:**

Multidose drug dispensing (MDD) is an adherence aid that provides patients with machine-dispensed medicines in disposable unit bags, usually for a 14 day period. Previous studies have suggested that the quality of prescribing, with time, is lower for MDD users, compared to patients receiving prescriptions dispensed as usual. This study aimed to examine the quality of prescribing to Norwegian elderly home care service patients receiving MDD.

**Methods:**

A cross-sectional study comprising 45,593 MDD patients aged ≥70 years was performed. The proportion of potentially inappropriate medications (PIMs) was assessed using the Norwegian General Practice Criteria, and drug-drug interactions (DDI) were investigated using the Norwegian Medicines Agency database.

**Results:**

On average, patients were prescribed 10.6 drugs (SD = 5.0), of which 6.1 were dispensed via MDD. Men used on average fewer drugs than women (10.7 vs 11.1), Twenty-seven percent of patients used at least one PIM. Concomitant use of three or more psychotropic drugs (10.8%), and prescribing of diazepam (6.4%) was the most commonly identified inappropriate prescribing. DDIs affected 59% of the patients, however, only 2.7% had serious interactions. Women were more frequently exposed to both PIMs and DDIs than men, with an odds ratio of 1.50 (95% CI: 1.43–1.58) and 1.43 (95% CI: 1.37–1.50), respectively.

**Conclusions:**

Polypharmacy is common in elderly Norwegian patients using MDD. About one-fourth of the patients were exposed to PIMs, and over half were exposed to DDI.

## Background

Multidose drug dispensing (MDD) is an adherence aid that provides patients with machine-dispensed medicines in disposable plastic bags, usually for 14 days. The MDD bags are labeled with the patient’s name, the drug names and the time the medicines should be taken. Tablets and capsules can be dispensed via MDD, while medicines such as mixtures, inhalators, topical formulations, etc., are dispensed in their original packaging. However, all medicines are usually issued on the same prescription as the MDD medicines, including other regular medication, pro re nata (p.r.n) medications, and dietary supplements.

MDD users are typically elderly patients with difficulties handling and administering their medicines, in addition to using several regular medicines [1–3]. This puts them at high risk of experiencing side effects, medication errors, and other adverse drug reactions. Even though the scientific evidence of the effects of MDD is limited [4, 5], the system is recommended by the Norwegian health authorities for use by homecare service patients [6]. The number of patients receiving MDD in Norway has grown from 15,700 patients in 2006 to 90,500 in 2017 [7]. The majority (76%) receive home care services (HCS), 21% live in nursing homes and the remaining 5% are home-dwelling patients who get MDD at their local pharmacy [7]. Most medicines for chronic conditions are reimbursed by the Norwegian National Insurance Scheme. For patients in HCS, the municipality pay the additional costs for the packing of MDD. In 2017, 240,000 patients received HCS; 132,000 were > 67 years [8]. HCS provide nursing care, such as assistance with personal hygiene, wound care and help to administer medicines, as well as practical help like cleaning, food delivery, and laundry, allowing patients to be able to live at home for as long as possible before moving into a nursing home [9].

Different tools can be used to investigate potentially inappropriate medications (PIMs). The Norwegian General Practice (NORGEP) criteria assess the quality of prescribing to elderly patients in general practice [10]. According to these criteria, about one-third of the elderly Norwegian population is exposed to PIMs [11]. MDD users are, however, more likely to be exposed to PIMs than patients using ordinary dispensing [12–14]. A Norwegian study examined the quality of prescribing to patients receiving MDD in 2009, shortly after the MDD system was established in Norway, and found a prevalence of PIMs of 26% [2]. However, this study was carried out using an incomplete medication list.

In Norway, over 90% of prescriptions are electronic [15], however, the MDD prescriptions are still paper-based. The MDD prescriptions have to be faxed to the pharmacy, and GPs find the multidose system more time consuming than electronic prescribing [16, 17]. As a consequence, there are concerns that the MDD system might lead to the GP making fewer changes in the patients’ prescribed medicines, and increase the risk of medication errors in the transition between primary and secondary care, as shown in Sweden [18, 19]. Patients can also get duplicate prescriptions when GPs prescribe electronic prescriptions in addition to MDD prescriptions [20]. There is, however, an electronic MDD prescribing system in the making, where the prescribing procedure will be the same for MDD prescriptions and ordinary prescriptions. This system is expected to improve the prescribing quality for MDD patients [15, 20].

This study aims to assess the prevalence of potentially inappropriate medication use among elderly patients receiving MDD in Norway, before the implementation of an electronic MDD prescribing system.

## Methods

### Study design and sample

We conducted a cross-sectional study using the medication lists from MDD patients in Norway, containing medicines prescribed in June 2018. The MDD supplier delivered about 90% of all MDDs in Norway at the time of the study, and provided anonymous study data for all patients in their system, containing age, gender and care setting (home dwelling, home care service, or nursing home). From the medication lists, details of drug names, strength, formulation, ATC code [21], dosage schedule and dispensing type (regular drugs dispensed via MDD, regular drugs not dispensed via MDD, or p.r.n medication) were obtained. The original dataset consisted of 87,519 patients and 859,642 medicines. As the NORGEP-criteria are applicable for elderly ≥70 years old, patients under the age of 70 were excluded. Self-pay patients and patients in nursing homes were also excluded because their medication lists are usually incomplete, only containing medicines dispensed as MDD, and no other regular medications and p.r.n. medications.

### Outcome measures

Each patient’s drug list was systematically screened for drug-drug interactions (DDIs) using the Norwegian Electronic Prescription Support System where DDIs are classified as either *(A) No action necessary*, *(B) Precautions should be taken*, or *(C) Should be avoided* [22]. Our data were screened for interactions of types B and C.

The PIMs were analysed employing the NORGEP criteria, a validated tool based on the updated American Beers Criteria [23] adapted to the Norwegian formulary, Swedish recommendations [24], and other literature [25]. The NORGEP criteria consists of 21 single substances and 15 drug combinations to be avoided in patients ≥70 years old (Table 1). Seven of 36 criteria were handled separately in the analysis: Antibiotics are usually prescribed for short periods, and therefore not listed on MDD prescriptions. Four NORGEP criteria on antibiotics (criterion 23, 24, 30, 35) were thus excluded from our analysis. One criterion (No 36) concerns concomitant prescription of three or more psychotropic medicines. In the analysis for this criterion, psychotropic medications had to be listed as *regular use* to be included, whereas p.r.n. psychotropic medications were excluded. For the two NORGEP criteria on overuse (no. 12,13), we could only analyse medicines dispensed via MDD and not as other regular medicines or p.r.n., because the dosing schedule was only available for the MDD medication. For the remaining 29 criteria, the criterion would be met if the medication was present in the list, regardless of whether it was listed as regular or p.r.n.

**Table 1:** Prevalence of potentially inappropriate medications in elderly (≥70 years) MDD users in Norway

	**Study population** **(*n*** **= 45,593)**		**Female** **(*n*** **= 30,090)**		**Male** **(*n*** **= 15,503)**		**Age 70–79** **(*n*** **= 11,435)**		**Age 80–89** **(*n*** **= 21,633)**		**Age 90+** **(*n*** **= 12,525)**	
**NORGEP single substance criteria**	**n**	**‰**	**n**	**‰**	**n**	**‰**	**n**	**‰**	**n**	**‰**	**n**	**‰**
1. Amitriptyline	848	19	639	21	209	13	312	27	381	18	155	12
2. Doxepin	61	1	44	1	17	1	17	1	25	1	19	2
3. Clomipramine	74	2	64	2	10	1	31	3	33	2	10	1
4. Trimipramine	162	4	120	4	42	3	60	5	69	3	33	3
5. Chlorpromazine (withdrawn from Norw. market 2007)	5	0	5	0	0	0	4	0	1	0	0	0
6. Chlorprothixene	387	8	234	8	153	10	222	19	130	6	35	3
7. Levomepromazine	391	9	255	8	136	9	195	17	140	6	56	4
8. Prochlorperazine	288	6	230	8	58	4	59	5	133	6	96	8
9. Diazepam	2911	64	2175	72	736	47	1011	88	1269	59	631	50
10. trazepam	839	18	595	20	244	16	227	20	342	16	270	22
11. Flunitrazepam	15	0	12	0	3	0	7	1	4	0	4	0
12. Oxazepam > 30 mg/24 h	335	7	253	8	82	5	179	16	116	5	40	3
13. Zopiclone > 7.5 mg/24 h	142	3	98	3	44	3	65	6	52	2	25	2
14. Carisoprodol (withdrawn from Norw. market 2008)	1	0	0	0	1	0	0	0	1	0	0	0
15. Dextropropoxyphene (withdrawn from Norw. market 2009)	0	0	0	0	0	0	0	0	0	0	0	0
16. Theophylline	107	2	68	2	39	3	45	4	50	2	12	1
17. Sotalol	210	5	133	4	77	5	30	3	116	5	64	5
18. Dexchlorpheniramine	64	1	42	1	22	1	20	2	26	1	18	1
19. Promethazine	85	2	54	2	31	2	31	3	35	2	19	2
20. Hydroxyzine	766	17	472	16	294	19	252	22	306	14	208	17
21. Alimemazine/ trimeprazine	964	21	634	21	330	21	419	37	395	18	150	12
**NORGEP combination criteria**												
22. Warfarin + NSAID	36	1	23	1	13	1	8	1	19	1	9	1
23. Warfarin + ofloxacin/ ciprofloxacin	NOT ANALYZED: ANTIBIOTICS ARE NOT INCLUDED ON THE MDD PRESCRIPTIONS											
24. Warfarin + erythromycin/ clarithromycin	NOT ANALYZED: ANTIBIOTICS ARE NOT INCLUDED ON THE MDD PRESCRIPTIONS											
25. Warfarin + SSRI	348	8	229	8	119	8	83	7	183	8	82	7
26. NSAID + ACE inhibitor/ARB	573	13	407	14	166	11	195	17	270	12	108	9
27. NSAID + diuretics	633	14	454	15	179	12	181	16	272	13	180	14
28. NSAID + glucocorticoids	206	5	160	5	46	3	68	6	98	5	40	3
29. NSAID + SSRI	370	8	309	10	61	4	139	12	174	8	57	5
30. Erythromycin/ clarithromycin + statin	NOT ANALYZED: ANTIBIOTICS ARE NOT INCLUDED ON THE MDD PRESCRIPTIONS											
31. ACE inhibitor+ potassium /potassium-sparing diuretic	1035	23	625	21	410	26	324	28	457	21	254	20
32. Fluoxetine/ fluvoxamine + TCA	3	0	2	0	1	0	2	0	1	0	0	0
33. Beta blocker + cardioselective calcium antagonist	147	3	107	4	40	3	28	2	67	3	52	4
34. Diltiazem + lovastatin/ simvastatin	34	1	22	1	12	1	13	1	15	1	6	0
35. Erythromycin/clarithromycin + carbamazepine	NOT ANALYZED: ANTIBIOTICS ARE NOT INCLUDED ON THE MDD PRESCRIPTIONS											
36. Concomitant prescription of three or more drugs from the groups centrally acting analgesics, antipsychotics, antidepressants, and/or benzodiazepines	4906	108	3775	125	1131	73	1959	171	2047	95	900	72

### Statistical analysis

Statistical analysis was performed using Stata/MP 15. Means and standard deviation were used to describe the sample characteristics, and student’s t-test was applied to compare means. Binary logistic regression was used to assess the relationships between inappropriate mediations (yes/no) for each patient, drug-drug-interactions (yes/no), and gender, age, and number of medicines. For the analysis, age was categorized into 10-year age intervals.

### Ethics

The study was approved by the Data Protection Officer at the University Hospital of North Norway. All data were anonymous and deemed not to need approval by the Regional Committees for Medical and Health Research Ethics.

## Results

**Fig. 1:**
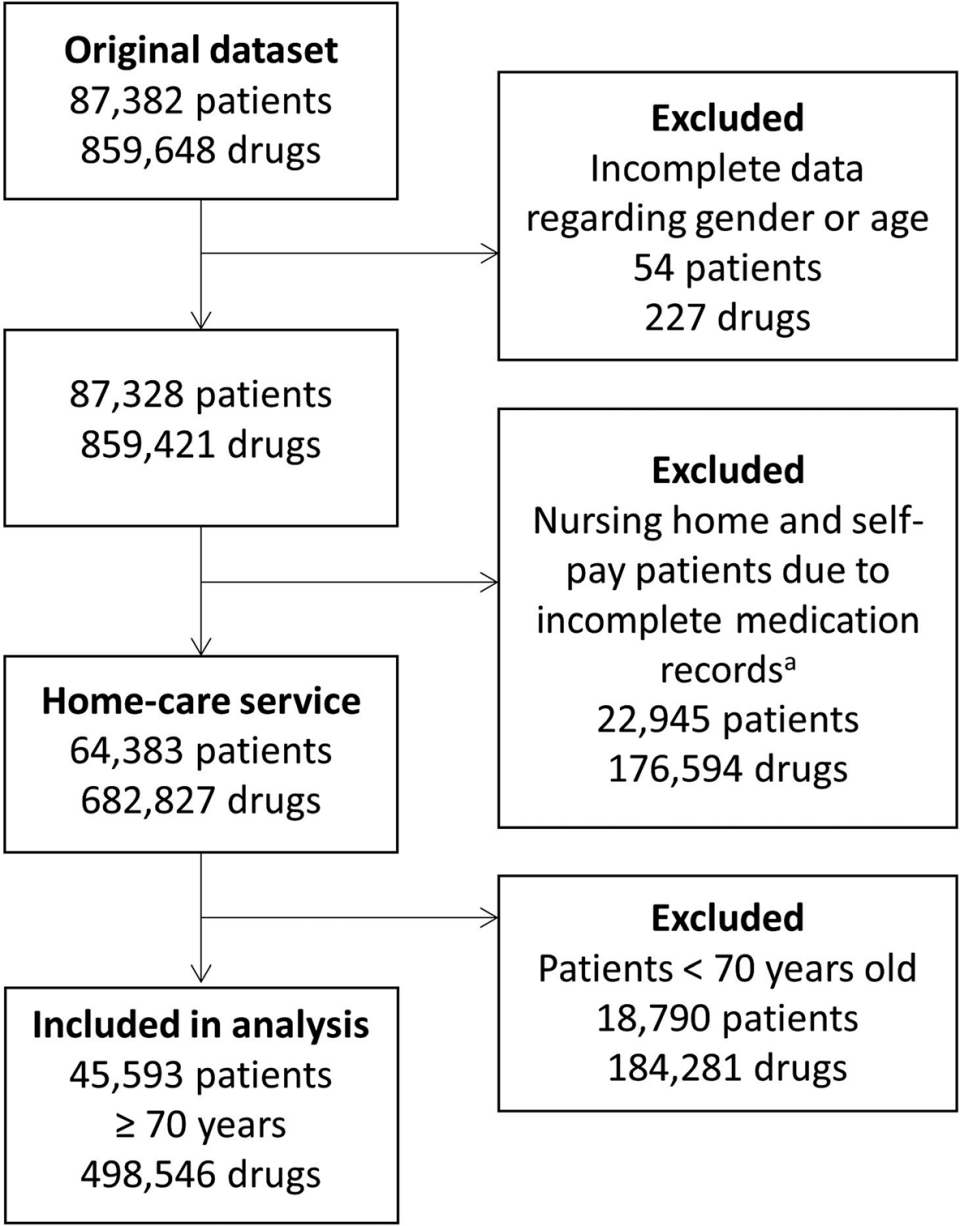
Patients using multidose drug dispensing in Norway in 2018: Exclusion flow chart of cases. a: Lists from the nursing home and self-pay patients only contain medicines dispensed as MDD, and not other regular medicines and p.r.n medication

As shown in Fig. 1, after exclusions the final analysis included 45,593 patients, i.e. approximately one-third of the patients ≥70 years old receiving home care services in Norway [8]. The study population characteristics are shown in Table 2. The mean number of regular medications was 8.2 (median = 8), of which 6.1 (median = 6) were dispensed as MDD. The mean number of total prescribed medicines was 10.6 (median = 10). In total 85% used 5 or more medicines regularly and 33% used 10 or more medicines. In addition, 20 % of the patients used dietary supplements. The mean age was 84.7 (SD = 7.3). Women were on average older than men (85.5 vs. 83.1, *p* < 0.001), and used a higher number of drugs (11.1 vs 10.7, p < 0.001). Drugs for the cardiovascular and nervous-system were the most frequently used drug groups. The most commonly prescribed therapeutic subgroups were antithrombotics (70% of patients), non-opioid analgesics (58%), beta-blockers (47%), lipid-modifying drugs (41%) and hypnotics/sedatives (39%) (Table 3).

**Table 2:** Study population characteristics and drug use (*N* = 45,593)

	**Study population**		**Regular drugs** **Dispensed as MDD**		**Regular drugs** **Not dispensed as MDD**		**P.r.n drugs**		**Total number of drugs**	
	**n**	**(%)**	**mean**	**(SD)**	**mean**	**(SD)**	**mean**	**(SD)**	**mean**	(SD)
**Total**	45,593	(100)	6.3	(2.8)	2.2	(2.0)	2.4	(2.3)	10.9	(5.0)
**Age**										
70–79	11,435	(25)	6.9	(3.1)	2.3	(2.1)	2.4	(2.4)	11.6	(5.5)
80–89	21,633	(47)	6.4	(2.8)	2.2	(1.9)	2.4	(2.3)	11.0	(4.9)
90+	12,525	(27)	5.7	(2.6)	2.1	(1.9)	2.4	(2.2)	10.3	(4.6)
**Gender**										
Female	30,090	(66)	6.4	(2.9)	2.5	(2.3)	2.6	(1.9)	11.1	(5.1)
Male	15,503	(34)	6.2	(2.8)	2.2	(2.2)	2.2	(2.0)	10.7	(4.9)

**Table 3:** The 25 most frequently used drug groups among MDD patients with home care services (*N* = 45,593)

**ATC level 3**	**Therapeutic drug group**	**Regular drugs Dispensed as MDD**		**Regular drugs Not dispensed as MDD**		**P.r.n** **medications**		**Drug use** **female(*n* = 30,090)**		**Drug use** **male** ***(n* = 15,503)**	
		**n**	**(%)**	**n**	**(%)**	**n**	**(%)**	n	(%)	n	(%)
B01A	Antithrombotic agents	27,477	(60)	5744	(13)	65	(0)	19,845	(66)	12,004	(77)
N02B	Other analgesics and antipyretics	11,393	(25)	1448	(3)	16,024	(35)	18,829	(63)	7846	(51)
C07A	Beta blocking agents	21,522	(47)	79	(0)	174	(0)	13,796	(46)	7709	(50)
C10A	Lipid modifying agents, plain	18,545	(41)	133	(0)	10	(0)	10,911	(36)	7643	(49)
N05C	Hypnotics and sedatives	10,485	(23)	495	(1)	7426	(16)	12,841	(43)	5984	(39)
A02B	Drugs for peptic ulcer and gastro-oesophageal reflux disease	16,036	(35)	289	(1)	1599	(4)	11,371	(38)	5266	(34)
C03C	High-ceiling diuretics	13,832	(30)	136	(0)	1992	(4)	9962	(33)	4939	(32)
A06A	Drugs for constipation	578	(1)	8926	(20)	7918	(17)	9502	(32)	4727	(30)
N02A	Opioids	3771	(8)	3758	(8)	9375	(21)	9869	(33)	3695	(24)
N06A	Antidepressants	11,564	(25)	429	(1)	178	(0)	8807	(29)	3377	(22)
N05B	Anxiolytics	3999	(9)	219	(0)	7743	(17)	8205	(27)	3171	(20)
A12A	Calcium	10,328	(23)	336	(1)	19	(0)	8821	(29)	3155	(20)
C08C	Selective calcium channel blockers with mainly vascular effects	9055	(20)	79	(0)	81	(0)	6269	(21)	3060	(20)
R03A	Adrenergics, inhalants	1	(0)	6809	(15)	4651	(10)	5542	(18)	2901	(19)
A11E	Vitamin B-complex, including combinations	8262	(18)	173	(0)	36	(0)	5072	(17)	2721	(18)
B03B	Vitamin B12 and folic acid	2968	(7)	5792	(13)	192	(0)	5573	(19)	2698	(17)
C09A	ACE inhibitors, plain	7569	(17)	37	(0)	1	(0)	4539	(15)	2644	(17)
C01D	Vasodilators used in cardiac diseases	3359	(7)	372	(1)	5997	(13)	4851	(16)	2498	(16)
H03A	Thyroid preparations	7050	(15)	29	(0)	2	(0)	5839	(19)	1981	(13)
C09C	Angiotensin II receptor blockers (ARBs), plain	6466	(14)	41	(0)	2	(0)	4547	(15)	1958	(13)
A10B	Blood glucose lowering drugs, excluding insulins	5734	(13)	229	(1)	12	(0)	3328	(11)	1927	(12)
R06A	Antihistamines for systemic use	3630	(8)	133	(0)	1984	(4)	3975	(13)	1872	(12)
D07A	Corticosteroids, plain	1	(0)	989	(2)	4504	(10)	3354	(11)	1844	(12)
A11C	Vitamin A and D, including combinations of the two	4709	(10)	209	(0)	61	(0)	3144	(10)	1819	(12)
R05C	Expectorants, excl. Combinations with cough suppressants	15	(0)	1187	(3)	3820	(8)	3003	(10)	1622	(10)

### Potentially inappropriate medications

**Fig. 2:**
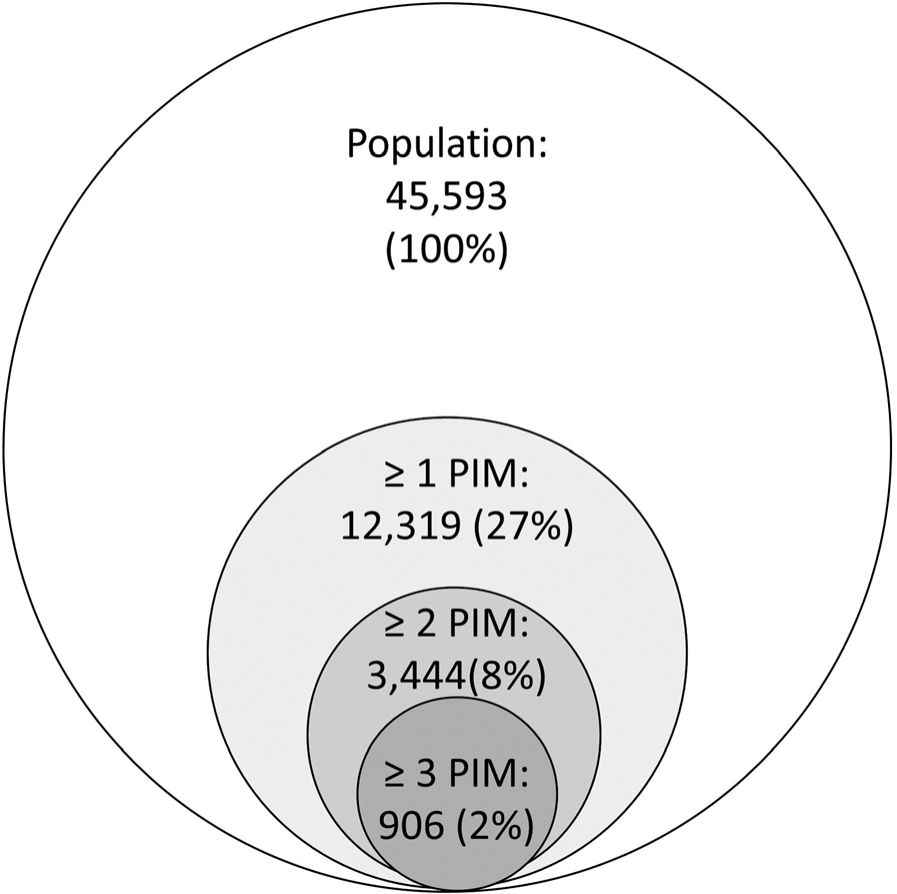
Prevalence of potentially inappropriate medications (PIMs) per patient

According to the NORGEP-criteria, 12,319 patients (27%) received one or more PIMs (Fig. 2). Table 1 shows the prevalence of the different PIMs. Concomitant use of three or more psychotropic and/or opioid drugs was the most prevalent PIM (10.8%), followed by prescribing of diazepam (6.4%). Criterion 1–8 and 18–21 concerns anticholinergic drugs; 3843 patients (8.4%) had one or more of those criteria. The number of PIMs was significantly correlated with the number of drugs prescribed (*p* < 0.001). After adjustment for age, women had a higher risk of PIMs (OR = 1.50, 95% CI: 1.43–1.58). The risk of PIMs decreased with patient age (Table 4).

**Table 4:** Factors associated with PIMs and DDIs in elderly (> 70 years) multidose users in Norway in 2018

**Study population**		**Potentially inappropriate medications (PIM)**				**Drug-drug interactions** **(DDI)**							
										**Type B**^**a**^		**Type C**^**a**^	
	**n**	**n**	**(%)**	**OR**	**(95% CI)**	**n**	**(%)**	**OR**	**(95% CI)**	**n**	**(%)**	**n**	**(%)**
**Age 70–79**	11,435	4072	(35.6)	1	(ref)	7227	(63.2)	1	(ref)	7171	(62.7)	493	(4.3)
**Age 80–89**	21,633	5449	(25.2)	0.61	(0.57–0.64)	13,000	(60.1)	0.90	(0.85–0.95)	12,935	(59.8)	543	(2.5)
**Age 90+**	12,525	2793	(22.3)	0.55	(0.52–0.59)	6785	(54.2)	0.76	(0.72–0.81)	6754	(53.9)	226	(1.8)
**No. of drugs**	45,593	NA		1.15^b^	(1.15–1-16)	NA		1.30^b^	(1.28–1.30)	NA		NA	
**Male**	15,503	8814	(22.6)	1	(ref)	8354	(53.9)	1	(ref)	8294	(53.5)	420	(2.7)
**Female**	30,090	3505	(29.3)	1.50	(1.43–1.58)	18,658	(62.0)	1.43	(1.37–1.50)	18,566	(61.7)	842	(2.8)

a: Type B = “Precautions should be taken”, Type C = “should be avoided”

^b^: Increase in odds for PIMs and DDIs for every one unit increase in the number of drugs

### Drug-drug interactions

**Fig. 3:**
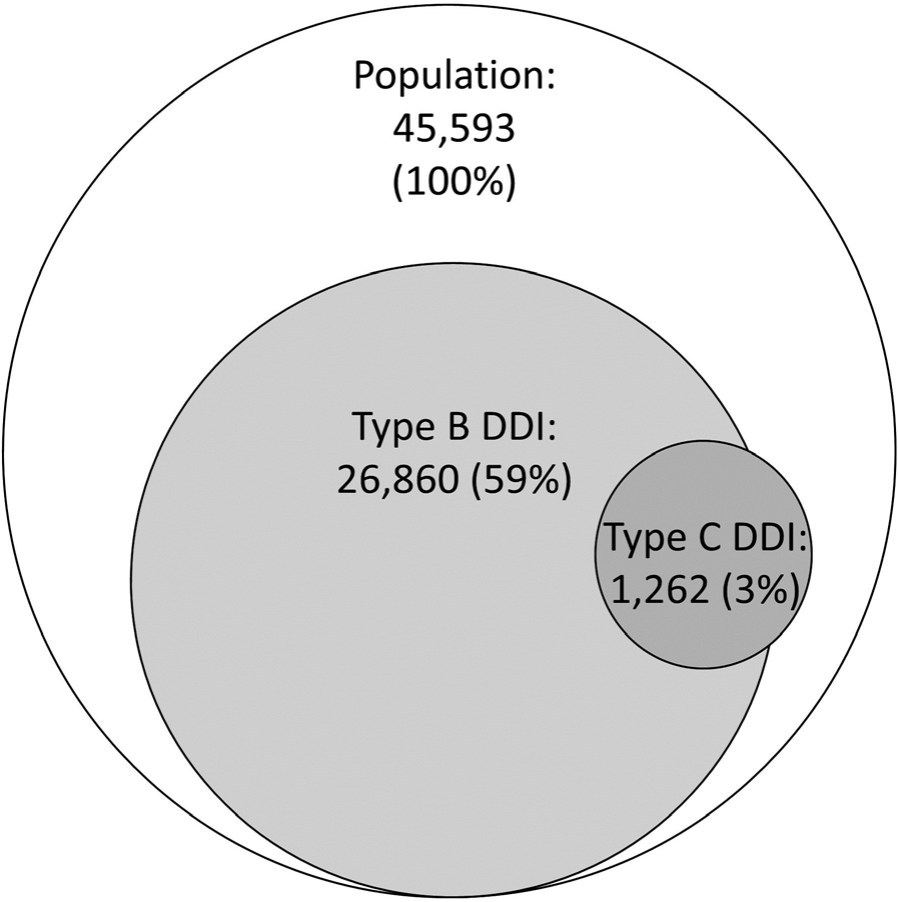
Prevalence of drug-drug interactions (DDIs) per patient. Type B =” Precautions should be taken”, Type C =” should be avoided”

The screening for DDI revealed 59,414 interactions in 27,012 (59%) of the patients. Of the total number of interactions, 97.7% were classified as “type B – precautions should be taken”, and 2.3% as “type C- should be avoided”. Figure 3 illustrates the number of patients with type B and C DDIs. DDIs increased with the number of prescribed drugs and decreased with patient age (Table 4). Women had a higher risk of DDIs than men (OR = 1.43, 95% CI: 1.37–1.50).

## Discussion

The medication use in elderly patients using MDD in home care services in Norway is high, with about one-third of the patients using 10 or more drugs regularly. Approximately one-fourth received potentially inappropriate medications, and over half was exposed to drug-drug interactions. Females had a higher risk than men to experience both PIMs and DDIs, and both PIMs and DDIs were positively correlated with the number of medicines prescribed and negatively associated with patient age.

### Drug utilization

The most commonly prescribed medication groups are the same in the MDD population as in the general elderly population not receiving MDD, however, the overall prevalence is higher in MDD patients (Table 3) [26]. Antithrombotic agents (70% in the present study vs. 47% general elderly population) and analgesics (58% vs. 25%) are more frequently prescribed in MDD patients than the general elderly population. Women were more frequently prescribed medications acting on the nervous system and less cardiovascular drugs compared to men. The MDD patients used on average 8.2 drugs regularly, of which 6.1 were dispensed as MDD. In addition, 2.4 drugs were listed as p.r.n. medications. This is a higher number of medicines compared to the Norwegian elderly community-dwelling patients [26, 27], and thus supports previous findings that MDD patients tend to use more medicines than patients with ordinary dispensing [1, 3, 13]. However, since our study population receives HCS, they also have greater care needs than the general population.

Polypharmacy has been associated with negative health outcomes such as falls, adverse drug reactions, hospitalization, and mortality [28, 29]. However, lack of proper adjustment for confounders has been mentioned as a challenge in these studies [28–30]. The recent ESTHER study found no independent association between polypharmacy and non-cancer mortality when adjusting for confounding by indication [31, 32]. The high prevalence of polypharmacy in our study might thus be a reflection of a high morbidity in the study population. One previous study has shown that introducing an MDD system increases the number of medicines prescribed [13], though another observed the same increase in the control group where MDD was not introduced [33].

### Potentially inappropriate medications

In our study, 27% of the patients had at least one PIM. A systematic review of PIMs found an estimated prevalence of 22.6% in European community-dwelling older adults [34]. The prevalence, however, varied greatly between the studies due to different quality indicators used and differences in the study populations included. Nyborg et al. used the NORGEP criteria and found a 34.8% prevalence of PIMs for the entire Norwegian home-dwelling elderly population [27]. This is higher than in our study, despite our study population using more medicines. An explanation is that we have excluded the criteria on the use of antibiotics. In addition, we used different data sources; Nyborg et al. used dispensed medicines while we used prescribed medicines (see strength and limitation).

Our prevalence of PIMs according to the NORGEP criteria (27%) is comparable with Halvorsen et al. (24.6%) a decade ago [2]. Though our prevalence (27%) is somewhat higher, this is expected since we have looked at the entire medication list of the patients, and not just the medicines dispensed as MDD. The prescribing quality for MDD patients over the past decade does not seem to have improved, despite increased focus on medication reviews and deprescribing [35, 36].

Concomitant use of three or more psychotropic drugs is a PIM in the NORGEP list, as this increases the risk of muscular weakness, falls, fractures and cognitive impairment [25]. Similar to our study, Halvorsen et al. found that this was the most prevalent PIM in MDD patients, with 9.0% meeting this criterion compared to 10.8% in our study. This is high compared to the general Norwegian elderly population, where the prevalence is 4.8% [27], however, it is lower in the Swedish studies of MDD patients, which found a prevalence of between 16.0 and 22.1% [3, 13, 14].

### Drug-drug interactions

The prevalence of DDIs in older patients vary greatly in the literature, from a few percent to almost 60% [2, 37, 38]. A prevalence of 59% as found in our study, is thus high. The majority of DDIs are “Type B – precautions should be taken” (Table 4). The suggested precautions for these DDIs include changing the ingestion time, increased monitoring of symptoms or side-effects and dose adjustments. The data in our study do not include information on whether precautions have actually been taken, however, the DDIs might not be clinically relevant if they have.

The prevalence of the most serious DDIs (type C) is more similar in our study and the literature. In our study, 2.7% of the patients had such interactions, while the prevalence is between 0.4 and 9.0% in previous studies of MDD patients [2, 3, 38, 39]. As the MDD prescriptions are systematically screened for DDIs using the same database we have used in this study, the GP is likely aware of these interactions at the time of the prescribing. Considering the low prevalence of the most serious DDIs we could thus question the clinical relevance of the interactions found in this study, as the doctor might already have judged the co-prescribing as necessary with no better alternatives available, and started appropriate monitoring of the patients.

### Predictors of PIMs and DDIs

We found that younger elderly (70–79 years) had a higher number of inappropriate drugs, a relationship confirmed by others [2, 39, 40]. However, Nyborg found that the age effect was not present in the multivariate analysis when in addition to age and gender, the number of prescribers was also included [27]. Information about the number of prescribers was, however not available for our study. Women having a higher risk of experiencing DDIs and PIMs, is also consistent with previous findings [2, 27, 38, 40, 41]. This is partly explained by the fact that women are more commonly prescribed sedatives, analgetics and anxiolytics (See Table 3) [41], and many of the PIMs are related to these drugs.

### Strengths and limitations

A major strength of this study is that it represents almost 90% of the MDD users in Norway. In addition, the medication lists include dietary supplements. This data gives comprehensive information on drug use for these patients. HCS patients and MDD-prescriptions cannot be specifically identified in the Norwegian Prescription Database [42]. In that sense, our data on medicines use is unique. There could still, however, be errors or omissions in the data. The patient can get prescriptions on antibiotics and other short-term treatments or buy over-the-counter medicines, which are not listed on the MDD prescription. Most Nordic countries have databases over dispensed prescriptions [43] while the medication list used in our study represents prescribed medications. Our data thus includes prescriptions issued by a physician but not filled (“primary non-compliance”), which makes comparison to other Nordic studies difficult.

As with all register-based studies, one cannot conclude if PIMs have led to actual drug-related problems for the patients. A recent study looking at patients with multimorbidity acutely admitted to the hospital, found that strict adherence to the NORGEP-criteria could have prevented 15% of the serious adverse drug reactions [44]. The NORGEP-criteria was published in 2009. Changes in both prescribing patterns and the Norwegian formulary have led to some of the items on the NORGEP list to be outdated (e.g. Table 1 shows that three drugs have been withdrawn from the Norwegian marked), and newer therapies which can be considered inappropriate for elderly have not been included. In addition, drug-specific criteria like the NORGEP-criteria do not capture all aspects of prescribing quality, as it, for example, does not address problems like under-prescribing like e.g. the START/STOPP criteria [35].

### Unfulfilled potential of the MDD system

Having all the patients’ medicines on the same prescription puts the pharmacist in a unique position to assess the prescribing and identify PIMs and DDIs. Having a complete overview over the patient’s medication use have been suggested as an explanation for why MDD patients seem to have fewer serious DDIs than patients with ordinary prescribing [14]. The systematic screening for DDIs for MDD patients might also explain the relatively low prevalence (2.7%) of serious DDIs in the present study. However, there still seems to be an unfulfilled potential of using the MDD system to systematically identify PIMs. The screening could be used to identify high-risk patients who could be targeted for interprofessional medication reviews which again could raise awareness of inappropriate prescribing. When the electronic prescribing system is implemented, this also opens possibilities for the pharmacist to give direct feedback to the prescriber when problems are detected.

Further research is needed to explore whether high overall medication use is a result of the MDD system in itself, or whether it is due to differences in patient characteristics for patients with ordinary prescriptions compared to MDD.

## Conclusions

This study suggests that potentially inappropriate prescribing is common in elderly patients receiving MDD in Norway, as about one-fourth of the patients were exposed to PIMs, and over half were exposed to DDIs. However, previous studies suggest that both PIMs and DDIs are common also in patients not receiving MDD. Comparing our results to previous Norwegian studies, we do not find the same difference in prescribing quality between patients with MDD and patients with ordinary prescribing, as is shown in Sweden. However, we see that the overall drug consumption in MDD patients is higher than the general population, with about one third being prescribed 10 or more drugs regularly. In addition, there is more frequent co-prescribing of psychotropic and opioid drugs in MDD patients.

## Data Availability

The data that support the findings of this study are provided by Apotek 1 Gruppen AS and are not publicly available. Data are however available from the corresponding authors upon reasonable request and with permission of Apotek 1 Gruppen AS.

## Abbreviations

MDD: Multidose drug dispensing
PIM: Potentially inappropriate medication
DDI: Drug-drug interactions
HCS: Home care service
NORGEP-criteria: The Norwegian General Practice-criteria
